# Silicone-Induced Foreign Body Reaction: An Unusual Differential Diagnosis of Posterolateral Hip Pain

**DOI:** 10.1155/2018/1802794

**Published:** 2018-07-08

**Authors:** Karishma Ramsubeik, Omar Tolaymat, Gurjit Kaeley

**Affiliations:** University of Florida, Jacksonville, FL, USA

## Abstract

**Background:**

Silicone injection is commonly used for soft tissue augmentation for esthetic purposes. It is not without complications.

**Case presentation:**

We present a case of a 31-year-old woman presenting with refractory left lateral hip pain. Magnetic resonance imaging of the patient's pelvis revealed innumerable small low signal foci throughout the gluteus maximus and overlying subcutaneous fat bilaterally consistent with injectable material, possibly silicone.

**Conclusions:**

This case report emphasizes that silicone-induced granulomatosis must be considered in the differential diagnosis of hip pain when evaluating a patient who has had access to plastic surgery or clandestine operators.

## 1. Background

Injectable silicone use for soft tissue augmentation dates as far back as 1940 [1]. Although in the United States of America, liquid injectable silicone (LIS) is FDA-approved only for intraocular use, it is used by both physicians and illegally by nonmedical personnel. The use for soft tissue augmentation remains controversial because of the possibility of severe reactions. We report a case of a 31-year-old woman presenting with refractory left lateral hip pain due to silicone-induced granulomatosis.

## 2. Case Presentation

A 31-year-old female presented with a five-year history of left lateral hip pain. She was initially seen at an outside facility and was diagnosed with trochanteric bursitis. At that time, she received a non-X-ray-guided steroid injection to the trochanteric bursa, which resulted in worsening of her pain. On presentation to our clinic, the pain was described as dull, 4/10, alleviated by rest and aggravated by movement. She denied joint swelling or erythema.

Her physical exam revealed normal gait, station, and full-range of movement of the left hip. There was no hip joint swelling, tenderness, or erythema. However, point tenderness over the left lateral thigh was elicited.

Laboratory studies revealed normal erythrocyte sedimentation rate (ESR) and c-reactive protein (CRP). X-ray of bilateral hips did not reveal any abnormality.

**Figure 1:**
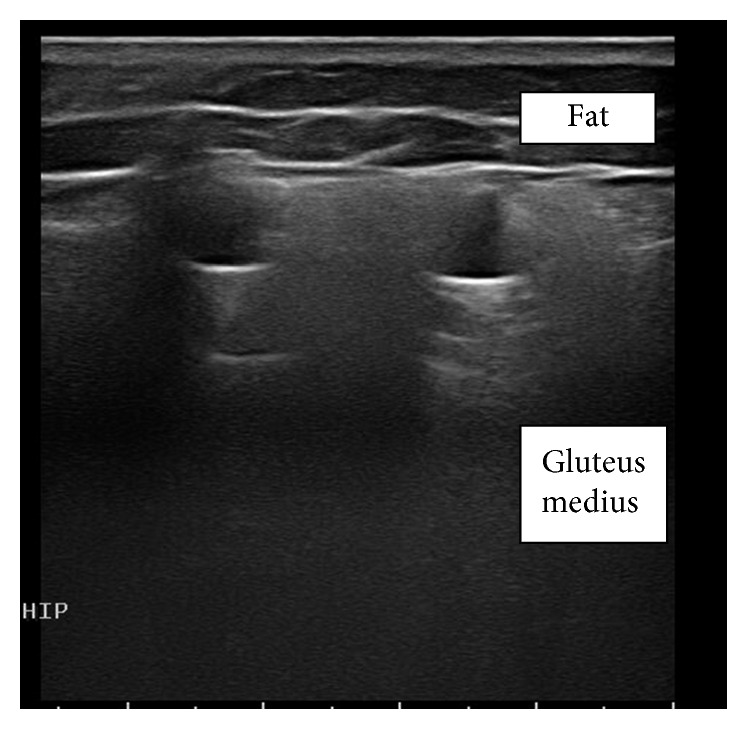
Ultrasound of the left hip showing dense hypoechogenicty of the gluteus medius with loss of normal echotexture and hyperechoic appearance as well as several anechoic areas posteriorly.

A musculoskeletal ultrasound (Figure 1) of the left lateral and posterior hip was performed. This showed normal gluteus miminus. However, there was dense hypoechogenicty of the gluteus medius with loss of normal echotexture. Posteriorly, there was a hyperechoic appearance as well as several anechoic areas. By probe palpation, tenderness correlated to the hyperechoic areas over the gluteus medius. The right lateral hip had similar but less prominent findings with the gluteus medius being the most affected. Further history was obtained which revealed that the patient had undergone silicone injections abroad, in the past.

**Figure 2:**
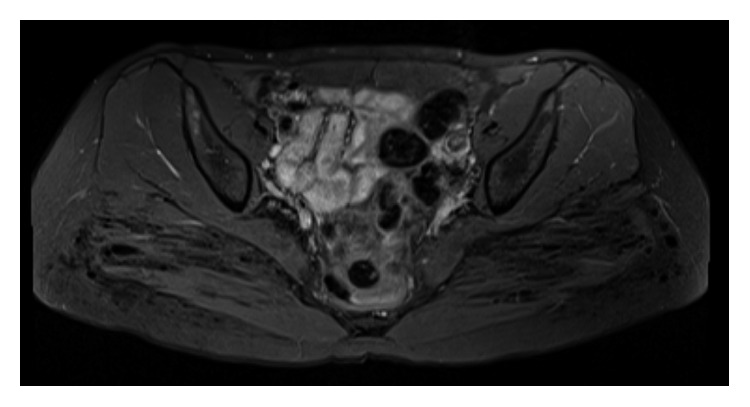
MRI of pelvis showing innumerable small low signal foci throughout the gluteus maximus and overlying subcutaneous fat bilaterally.

Since deeper tissues could not be visualized due to artifacts, magnetic resonance imaging (MRI) was ordered. MRI of the patient's pelvis with contrast (Figure 2) was obtained. This showed innumerable small low signal foci throughout the gluteus maximus and overlying subcutaneous fat bilaterally consistent with injectable material, possibly silicone.

## 3. Discussion

The use of injectable silicone for soft tissue augmentation remains controversial because of the possibility of severe reactions. Local injection side effects include mild erythema and edema. These are usually self-limited and resolve spontaneously. Silicone can also track along tissue lines migrating to distant sites. There have also been reports of granuloma formation presenting as edematous, inflamed nodules. Liquid injectable silicone is thought be relatively safe as long as medical grade silicone and limited volumes are used per session, and a microdroplet serial puncture technique is performed. However, no clear evidence supports this [1].

Gluteal injections of liquid silicone have similar local complications to other injection sites. In a retrospective case series of industrial grade, liquid silicone injections two out of 12 patients had bilateral buttock injections. Both patients had early complications, defined as <30 days, and included local erythema, edema, and pain. One of the two patients also had necrosis and abscess formation [2]. In a case report, filler migration to the bilateral lower extremities caused induration and pain after two injections to the bilateral buttock of unknown composition were performed 11 years prior. MRI showed migration of infiltrative material to bilateral lower extremities, and pathology was consistent with what was most likely silicone [3]. Silicone-induced gluteal granulomas were seen in two case reports: the first after one year of injection in an outpatient clinic in the Dominican Republic [4] and the other after injections abroad [5].

Systemic complications of gluteal silicone injections have been documented as well. A clandestine liquid silicone injection in the hip and buttock caused multisystem organ failures including respiratory failure. Multiple subacute white brain matter infarcts were also found to be consistent with embolization on histology [6]. Another case report described diffuse alveolar hemorrhage causing hypoxic respiratory failure requiring extracorporeal membrane oxygenation from silicone embolism after medically unsupervised silicone injection into the gluteal region [7].

The differential diagnosis of hip pain is broad. It is helpful to localize hip pain based on the anatomical region: anterior/groin, posterior, and lateral hip pain. Lateral hip pain occurs in 10–25% of the population. Greater trochanteric pain syndrome is a diverse clinical entity caused by different underlying conditions, such as trochanteric bursitis, gluteus medius/minimus tears, and iliotibial band thickening [8].

Trochanteric bursitis is part of the lateral hip pain syndrome differential diagnosis. It is a clinical diagnosis. Most of the time, imaging is not used to make the diagnosis. In this case, outside image guided-trochanteric bursa injection did not help, and musculoskeletal ultrasound was thus requested.

In our patient, left lateral hip pain was the presenting symptom. The history was nonspecific but notable for worsening of pain after prior steroid injection. On physical exam, there was point tenderness over the left greater trochanter suggesting bursitis. Our patient was reluctant to have steroid injection without further diagnostic testing. Neither musculoskeletal ultrasound nor MRI showed bursitis but did show evidence of injected silicone.

Silicone injections cause a factitial panniculitis [9]. This has a characteristic histopathological pattern of a lobular panniculitis with small droplets of silicone oil found within the vacuoles and giant multinucleated cells [10, 11]. Biopsy was not performed in this patient.

The treatment of silicone granulomas can be difficult. Tetracyline antibiotics [12, 13], steroids [12, 14], and etanercept [15, 16] have all been described in the literature with varying degrees of success. Surgical excision can be considered if the granuloma is localized and well circumscribed, but this is rarely the case [17].

## 4. Conclusion

In evaluating a young patient presenting with lateral hip pain who has had access to plastic surgery or clandestine operators, consider silicone-induced foreign body reactions.

## Abbreviations

CRP: C-reactive protein
ESR: Erythrocyte sedimentation rate
MRI: Magnetic resonance imaging.
